# A Comprehensive Review of US FDA-Approved Immune Checkpoint Inhibitors in Urothelial Carcinoma

**DOI:** 10.1155/2017/6940546

**Published:** 2017-12-10

**Authors:** Fu-Shun Hsu, Chun-Hung Su, Kou-How Huang

**Affiliations:** ^1^Department of Urology, New Taipei City Hospital, New Taipei City, Taiwan; ^2^Graduate Institute of Clinical Medicine, National Taiwan University College of Medicine, No. 7 Chung San South Road, Taipei 10002, Taiwan; ^3^Genomics Research Center, Academia Sinica, No. 128, Academia Rd., Sec. 2, Taipei 11529, Taiwan; ^4^Department of Urology, National Taiwan University Hospital, No. 1, Jen-Ai Rd., Sec. 1, Taipei 10051, Taiwan

## Abstract

Few effective treatment options are available for patients with advanced or metastatic urothelial carcinoma (UC) after unsuccessful first-line platinum-based chemotherapy. To date, immune checkpoint inhibitors are novel therapeutic agents for UC treatment. From May 2016 to May 2017, five anti-PD-1/PD-L1 monoclonal antibodies received accelerated or regular approval from the US Food and Drug Administration (FDA) for the treatment of patients with locally advanced or metastatic UC. The present comprehensive review presents the background information of these five US FDA-approved anticancer agents to provide a basic but concise understanding of these agents for advanced studies. We summarize their immune checkpoint mechanisms, clinical efficacy, recommended usage protocols, adverse events, and the limitations of the PD-L1 biomarker assays.

## 1. **Introduction**

Urothelial carcinoma (UC) is one of the top ten leading causes of cancer death worldwide. UC tumorigenesis is thought to be associated with environmental carcinogenic exposure such as cigarette smoking and chemical exposure [1]. The pathological sites of UC include the renal pelvis and ureter in the upper tract as well as bladder and urethra in the lower tract. Among them, the bladder is the most common site of UC occurrence. In the United States, it was estimated that 79,030 new cases and 16,870 deaths were due to bladder UC in 2017 [2].

Bacillus Calmette–Guérin (BCG), an attenuated live strain of *Mycobacterium bovis*, has been used for treatment in patients with nonmuscle invasive bladder UC since the 1990s. The benefits from intravesical BCG instillation have been proven, including lowering the risk of disease recurrence and disease progression [3]. BCG is the standard treatment for patients with nonmuscle invasive bladder UC following transurethral resection of bladder tumors for decades, but underlying mechanism of its antitumor effect remains unclear. BCG induces a local inflammatory response and recruits immune cells to destroy tumor cells and, therefore, plays a vital role in bladder cancer immunotherapy. There are some limitations in BCG treatment, including high failure rate and risk of systemic infection.

Cisplatin-based systemic chemotherapy remains the mainstay of treatment in patients with metastatic UC [4]. There are still 30% to 50% of advanced UC cases that are not responsive to cisplatin-based chemotherapy. Although some new chemotherapy regimens have been developed, the prognosis for patients with metastatic UC remains poor [5]. Other limiting factors associated with standard regimen are the substantial toxicity and patients' physical conditions [6]. Treatment-related deaths occurred in 2% to 4% of patients, especially in the elderly [4, 7]. The median overall survival (OS) of patients with metastatic UC who received first-line platinum-based chemotherapy ranges from 12 to 15 months, and only approximately 5% of these patients have a 5-year survival [8–10]. The systemic salvage therapy for patients with advanced UC lasts only 6 to 8 months [11]. Unlike patients with other cancers, such as non-small-cell lung cancer (NSCLC) [12–14], breast cancer [15, 16], and leukemia [17], who can benefit from many targeted agents, including small molecule inhibitors or anticancer antibodies, patients with UC are still awaiting effective targeted drug treatments. Therefore, there is an urgent need to develop a novel therapy to improve therapeutic efficacy and patient survival or to reduce side effects for patients with locally advanced or metastatic UC.

## 2. Immune Checkpoint Therapy

**Figure 1:**
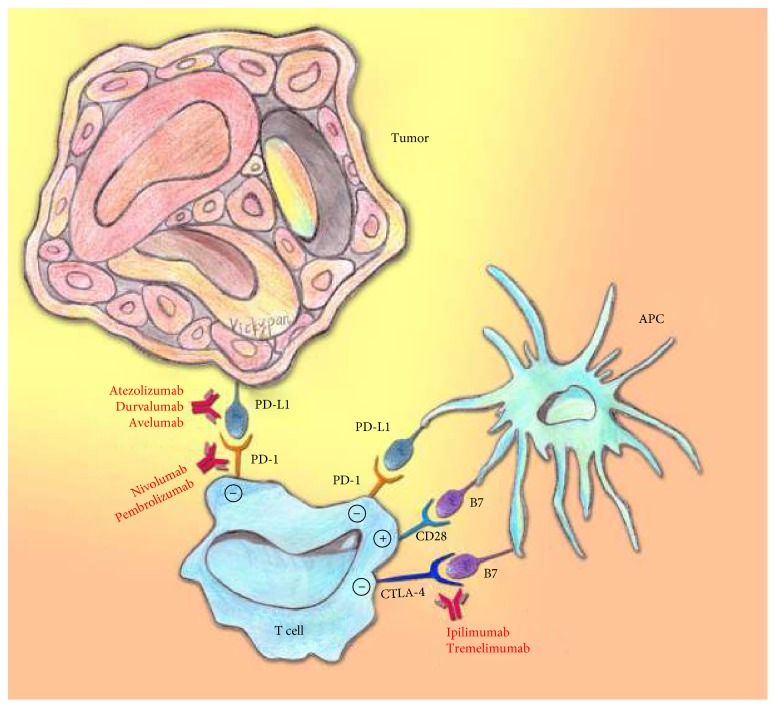
Illustration of anticheckpoint immunotherapy. The immune system is maintained and functions in homeostasis. Once CD28 binds to its ligand, B7, on the surface of antigen-presenting cells (APCs), T cell proliferation is activated to enhance immunity. On the other hand, cytotoxic T lymphocyte antigen-4 (CTLA-4) and programmed death-1 (PD-1) on APCs or tumor cells transmit inhibitory signals, while binding to their ligands, B7 and PD-L1, respectively. In general, the immune cells could recognize tumor cells and then destroy them. However, the tumor cells can escape from the host antitumor activities by suppressing the activation of immune cells. The anticheckpoint antibodies are developed to block the inhibitory pathways and then restore T cell immunity against tumors.

The immune system defends the body from an invasion by foreign etiological agents. The presentation of antigens to T cells by antigen-presenting cells (APCs) is a critical process (Figure 1). Several protein molecules involved in the regulation of immune processes and for homeostatic maintenance of the immune system have been identified. CD28 was the first protein to be identified as a coreceptor that transmits stimulatory signals to T cells. After CD28 binds to its ligand, the B7 protein, on the surface of APCs, T cell proliferation is activated to enhance immunity (Figure 1). Cytotoxic T lymphocyte antigen-4 (CTLA-4) and programmed death-1 (PD-1) transmit inhibitory signals when bound to their ligands, B7-1/B7-2 and PD-L1 (B7-H1)/PD-L2 (B7-DC), respectively, on APCs or tumors (Figure 1). Such protein molecules involved in immune regulation are referred to as immune checkpoints. Typically, the immune system is capable of recognizing and destroying tumor cells; however, tumor cells can exploit the inhibitory mechanism and evade the host antitumor activity by suppressing the proliferation of immune cells, subsequently survive, and continue to proliferate.

Immune checkpoint inhibitors were developed to control immune escape tumors. The most widely studied immune checkpoint inhibitors are anti-CTLA-4, anti-PD-1, and anti-PD-L1 monoclonal antibodies [18–20] (Figure 1) which target the T cell regulatory pathways to augment antitumor immune responses [21]. These inhibitors have shown promising efficacy in melanoma [22], renal cell carcinoma [23], NSCLC [24, 25], and bladder UC [26]. As in the case with immunotherapy for other types of cancers, these drugs show limited response rate, but the efficacy in achieving long-lasting benefits for some patients has changed the paradigm of cancer treatment. Due to the milestone discovery of the role of PD-1 [27], PD-L1 [28], and CTLA-4 [29] in inhibiting carcinogenesis, the 2017 Warren Alpert Foundation Prize was awarded to Drs. T. Honjo, L. Chen, James P. Allison, and colleagues to honor their significant contributions in the field of cancer immunotherapy [29]. This review focuses on the FDA-approved PD-1 and PD-L1 inhibitors in urothelial carcinoma.

## 3. US Food and Drug Administration-Approved Immune Checkpoint Inhibitors in Urothelial Carcinoma

The first checkpoint inhibitor approved for bladder UC was atezolizumab (Tecentriq) in 2016, which was the second-line therapy for patients who had received platinum-based chemotherapy. Response rates were around 15% with median survival of 7.9 months [30–32]. Other approved drugs that followed atezolizumab include durvalumab (Imfinzi) [33–35], nivolumab (Opdivo) [36], and avelumab (Bavencio) [37], and the latest is pembrolizumab (Keytruda) [38, 39]. All these US FDA-approved agents were approved for the treatment of patients with locally advanced or metastatic UC who experienced disease progression during or after platinum-based chemotherapy, or within 12 months of neoadjuvant or adjuvant treatment with platinum-based chemotherapy [30–42]. The clinical efficacy, adverse events, and recommended usage of these drugs are as follows.

### 3.1. Efficacy

Atezolizumab was the first immune checkpoint inhibitor approved by the US FDA on May 18, 2016, for the treatment of patients who experienced unsuccessful first-line platinum-based chemotherapy (Table 1). Atezolizumab is a human IgG1k antibody against the PD-L1 checkpoint. The US FDA-accelerated approval was obtained by the results of the IMvigor-210 study, a multicenter, single-arm trial of 310 patients with UC [30–32]. The IMvigor-210 study stratified patients with UC by PD-L1 expression levels in the tumor-infiltrating immune cells. Patients with ≥5% of tumor-infiltrating immune cells stained by PD-L1 in the tumor were categorized as a PD-L1-positive group. In this study, a total of 100 (32%) and 210 (68%) patients were categorized into PD-L1 positive and PD-L1 negative, respectively. The trial excluded patients with a history of autoimmune diseases or those who required systemic immunosuppressive medications. All patients received 1200 mg of atezolizumab intravenously every 3 weeks. The efficacy was evaluated by Response Evaluation Criteria in Solid Tumors version 1.1 (RECIST v1.1). The objective response rate (ORR) of all patients was 14.8% (Table 2) [30, 31]. The median duration of response ranged from 2.1 to 13.8 months. The ORR for patients with PD-L1 positive versus those were negative was 26.0% and 9.5%, respectively (Table 2) [30, 31].

Durvalumab is a humanized IgG1k antibody also against the PD-L1 checkpoint (Table 1) [33]. The US FDA granted accelerated approval to durvalumab for the treatment of patients with advanced or metastatic UC on May 1, 2017. The approval was based on a single-arm study of patients with UC who had unsuccessful first-line platinum-based chemotherapy [33, 34]. Recently, the results from the durvalumab trial involving 191 patients with UC have been updated [35]. Durvalumab (10 mg/kg every 2 weeks) was administered to patients intravenously. The efficacy was assessed using RECIST v1.1 criteria. The median duration of response ranged from 0.9 to 19.9 months. The trial also stratified patients with UC by PD-L1 expression levels. The ORR was 17.8% for all patients (*n* = 191) and was 27.6% (*n* = 27) and 5.1% (*n* = 4) in PD-L1 high expression and low (or negative) expression groups, respectively. The median OS was 18.2 months for all patients and was 20.0 months and 8.1 months in PD-L1 high expression and low (or negative) expression groups, respectively (Table 2) [35].

Avelumab is a human IgG1 antibody against the PD-L1 checkpoint. Avelumab received US FDA-accelerated approval on May 9, 2017, based on the results of the open-label, single-arm, multicenter JAVELIN study (Table 1) [37]. Avelumab was approved for the treatment of patients with UC who had disease progression after first-line platinum-based chemotherapy. In the JAVELIN trial, patients received avelumab (10 mg/kg every 2 weeks) intravenously until disease progression or intolerable toxicity. Before avelumab administration, all patients received antihistamine and acetaminophen. The ORRs at 13-week (*n* = 30) and 6-month (*n* = 26) follow-ups were 13.3% and 16.1%, respectively. The median duration of response ranged from 1.4 to 17.4 months (Table 2) [37].

Nivolumab is a human IgG4 antibody against the PD-1 checkpoint. Based on a single-arm clinical study, CheckMate-275 [36], the US FDA granted accelerated approval to nivolumab on February 2, 2017, for the treatment of UC after unsuccessful first-line platinum-based chemotherapy (Table 1). Nivolumab was also the first immune checkpoint inhibitor approved in the European Union for UC treatment on June 4, 2017. In the CheckMate-275 trial, nivolumab was administered to 270 patients with UC (3 mg/kg every 2 weeks) until disease progression or intolerable toxicity. The ORR following RECIST criteria was 19.6%. Seven patients (2.6%) had complete responses, whereas 46 (17%) had a partial response. The median duration of response was 10.3 months, and the median overall survival (OS) was 8.7 months (Table 2) [36].

Pembrolizumab is a humanized IgG4 antibody against the PD-1 checkpoint. Pembrolizumab is the latest immune checkpoint inhibitor approved by the US FDA on May 18, 2017, for the treatment of patients with UC (Table 1). In addition to the approval of second-line indication, pembrolizumab also received US FDA-accelerated approval for first-line indication for UC treatment. The first- and second-line indications were approved based on KEYNOTE-052 [40] and KEYNOTE-045 [38, 39] trials, respectively. In the KEYNOTE-052 trial, 370 patients with UC who were not eligible for cisplatin-based chemotherapy were enrolled and administered with pembrolizumab (200 mg every 3 weeks). The median follow-up was 7.8 months, and the ORR was 28.6%. The median duration of response ranged from 1.4 to 17.8 months. In the KEYNOTE-045 trial, 542 patients with UC were randomly assigned to receive either pembrolizumab (200 mg every 3 weeks; *n* = 270) or the investigator's choice of a chemotherapy regimen (every 3 weeks, *n* = 272) [38]. This trial produced significant improvements in the median OS and ORRs in both pembrolizumab- and chemotherapy-treated groups. The median OS was 10.3 and 7.4 months in pembrolizumab- and chemotherapy-treated groups, respectively (hazard ratio: 0.73; 95% CI: 0.59–0.91; *p* = 0.004). The ORRs were 21% and 11% for pembrolizumab- and chemotherapy-treated groups, respectively (*p* = 0.002). However, no significant differences were observed in the progression-free survival between the two regimen groups (Table 2) [38, 39].

### 3.2. Adverse Events

Table 3 presents the adverse events of the five US FDA-approved PD-1/PD-L1 inhibitors for patients with UC [30–38, 40, 43–47]. The most common treatment-related adverse events observed in about 15–20% of treated patients include fatigue, decreased appetite, nausea, and musculoskeletal pain. Urinary tract infection was reported in patients treated with the three PD-L1 inhibitors. Constipation was observed in the atezolizumab-, durvalumab-, and pembrolizumab-treated groups. In addition, pyrexia and peripheral edema were reported in the atezolizumab- and durvalumab-treated groups, respectively. Furthermore, the pembrolizumab-treated group had pruritus and rash. Diarrhea is commonly seen in PD-L1- and durvalumab-treated patients.

In addition, immune-targeted agents that can cause dysimmune toxicities in any tissue but mainly affect the lung, liver, gut, endocrine glands, and skin caused immune-related adverse events (IRAEs) [48]. Although severe IRAEs are rare, once occurred, they can be life-threatening if managed inappropriately [49].  Table 3 lists the common IRAEs of checkpoint inhibitor-treated patients. All five checkpoint inhibitor-treated groups might have pneumonitis, hepatitis, colitis, and endocrinopathies (e.g., thyroid disease, adrenal insufficiency, hypophysitis, and type 1 diabetes). Nephritis and renal dysfunction were commonly observed in all drug-treated groups except the atezolizumab-treated group. Meningitis/encephalitis and dermatitis/rash were observed in the atezolizumab- and nivolumab-treated groups. Pancreatitis may also in the atezolizumab-treated group. Other details of the IRAEs caused by checkpoint inhibitors are described elsewhere [6, 43–47].

### 3.3. Recommended Usage

Table 1 presents the recommended usage of the US FDA-approved immune checkpoint inhibitors for UC treatment. These antibodies are administered intravenously. The recommended doses and schedules for atezolizumab, nivolumab, durvalumab, avelumab, and pembrolizumab are 1200 mg every 3 weeks, 240 mg every 2 weeks, 10 mg/kg every 2 weeks, 10 mg/kg over a 60-minute influx every 2 weeks, and 200 mg over a 30-minute influx every 3 weeks, respectively, until disease progression or intolerable toxicity [30–40].

## 4. Discussion

Although upper tract urothelial carcinoma (UTUC) was identified with molecular profiling approaches that were different from those for bladder UC [50], the immune checkpoint inhibitors performed with promising efficacy in both UTUC and bladder UC [51]. However, many concerns remain. For example, the exact mechanism underlying the dominant role of PD-L1 expression in the efficacy of anti-PD-1/PD-L1 antibodies remains unclear. Furthermore, the influence of patients' genetic backgrounds, particularly racial differences, warrants further investigation.

According to our review of the relevant literature, previous studies did not provide the nucleotide sequence or protein compositions of PD-1/PD-L1 immune checkpoints in patients with UC. The relationships between the antigen-binding sites (paratopes) on the therapeutic monoclonal antibody inhibitors and the antibody-binding sites (epitopes) on the checkpoint proteins remain unclear. Nonetheless, UC has been identified as the tumor with high and heterogeneous mutation burden [52]. The genetic characteristics affect the efficacy of anticancer agents. The observation on tyrosine kinase inhibitor (TKI) treatments for NSCLC demonstrated a paradigm shift on the associations between mutation type and drug efficacy; moreover, even a single site mutation could have a substantial influence on drug sensitivity or resistance [53]. There is an urgent need to identify a biomarker as a clinical outcome predictor for patients with UC who can benefit from the anti-PD-1/PD-L1 immunotherapy.

Currently, PD-L1 is regarded as a biomarker in PD-1/PD-L1 inhibitor trials [20, 54–56] trials although the exact role of PD-L1 expression in the therapeutic efficacy of PD-1/PD-L1 inhibitors remains controversial [57]. For clinical practice, PD-L1 expression level of patients with metastatic melanoma or NSCLC is typically examined to determine whether the patients are suitable for treatment of anti-PD-1/PD-L1 immunotherapy [58]. For patients with UC, VENTANA PD-L1 SP142 and SP263 assays were used to classify them into PD-L1-positive or PD-L1-negative cohorts in atezolizumab and durvalumab trials, respectively [30, 31, 33, 35]. Those trials indicated patients with higher PD-L1 expression exhibiting improved efficacy compared to those with lower PD-L1 expression. However, of the variations in techniques, platforms, diverse specimens, tumor and immune microenvironment and the positive cutoff of PD-L1 expression complicate the standardization of decision-making in clinical applications [57]. Therefore, the classification of PD-L1-positive and PD-L1-negative groups for cancer patients is usually defined dynamically based on different assays or cutoffs. Currently, we suggest using PD-L1 expression level for outcome assessments but not for patient selections. Hence, the optimization of biomarker assays to identify the ideal population for anti-PD-1/PD-L1 immunotherapy is crucial for clinical practice [57, 58]. Alternatively, stratifying patients with UC based on the epitope sequences of their checkpoints and then applying the subtypes of the epitopes to develop the corresponding anti-PD-1/PD-L1 antibodies may contribute to the optimization of personalized and precision medicine.

Additionally, these PD-1/PD-L1 inhibitors may exert synergistic effects with other anticancer agents to prolong patients' survival or reduce side effects.  Table 4 shows selected new or ongoing clinical studies of PD-1/PD-L1 inhibitors for the treatment of UC. Those interventions are monotherapy of PD-1/PD-L1 inhibitors or combination therapy with anti-CTLA-4 antibodies, chemotherapy agents, or radiotherapy. Some studies are designed to discover the relationships between biomarker and the efficacy of PD-1/PD-L1 inhibitors as well as the effect of difference dosage levels. Their results may provide new clues or strategies in winning the fight against UC in the future.

In this compact but comprehensive review, we summarized the background information of the five US FDA approved PD-1 and PD-L1 checkpoint inhibitors as well as elucidate their mechanism of actions (MOA). We outlined their drug efficacy, safety, and adverse events from the clinical trials of patients with UC. These therapeutic antibodies have shown promising results in their respective FDA-approved trials and have given new hope to those who are suffering from advanced or metastatic UC. Further large-scale clinical trials of checkpoint inhibitor will reveal the optimal administration of these drugs and allow more patients with UC to benefit from immunotherapy treatments.

## Figures and Tables

**Table 1:** Background information on US FDA-approved PD-1/PD-L1 inhibitors for the treatment of urothelial carcinoma.

Target	Generic name	Antibody class	Trade name	Development name(s)	Company	Recommended dose and schedule	Date of approval
PD-1	Nivolumab	Human IgG4	Opdivo	BMS-936558, MDX-1106, ONO-4538	Bristol-Myers Squibb Co.	240 mg, every 2 weeks	2 Feb. 2017
	Pembrolizumab	Humanized IgG4	Keytruda	MK-3475, lambrolizumab	Merck and Co. Inc.	200 mg, every 3 weeks	18 May 2017
PD-L1	Atezolizumab	Human IgG1k	Tecentriq	MPDL3280A, RG7446	Genentech Inc.	1200 mg, every 3 weeks	18 May 2016
	Durvalumab	Humanized IgG1k	Imfinzi	MEDI-4736	AstraZeneca UK Limited	10 mg/kg, every 2 weeks	1 May 2017
	Avelumab	Human IgG1	Bavencio	MSB0010718C, MSB0010682	EMD Serono Inc.	10 mg/kg, every 2 weeks	9 May 2017

All are for the patients with locally advanced or metastatic urothelial carcinoma and who has the prior platinum-based chemotherapy.

**Table 2:** Efficacy outcomes of all tested patients with urothelial carcinoma in US FDA-approved PD-1/PD-L1 inhibitor trials.

Inhibitor target	Treatment regimen	Trial code	NCT identifier	Trial phase	Patient number	ORR (95% CI)	DoR/month (range)	PFS/month (95% CI)	OS/month (95% CI)	Reference
PD-1	Nivolumab	CheckMate-275	NCT02387996	Phase 2	265	19.6% (15.1, 24.9)	10.3	2.0 (1.87, 2.63)	8.74 (6.05, NR)	[36]
	Pembrolizumab	KEYNOTE-045	NCT02256436	Phase 3	542	21% (16.4, 26.5)	—	2.1 (2.0, 2.2)	10.3 (8.0, 11.8)	[38, 39]
		KEYNOTE-052	NCT02335424	Phase 2	370	28.6% (24, 34)	NR (1.4+, 17.8+)	—	—	[40]
PD-L1	Atezolizumab	IMvigor-210	NCT02108652	Phase 2	310	14.8% (11.1, 19.3)	NR (2.1+, 13.8+)	2.1 (2.1, 2.1)	7.90 (6.6, 9.3)	[30, 31]
	Durvalumab	Study 1108	NCT01693562	Phase 1/2	191	17.8 (12.7, 24.0)	NR (0.9+, 19.9+)	1.5 (1.4, 1.9)	18.2 (8.1, NR)	[35]
	Avelumab	JAVELIN	NCT01772004	Phase 1	44	^∗^13.3% (9.1, 18.4) ^∗∗^16.1% (10.8, 22.8)	NR (1.4+, 17.4+)	2.9 (1.53, 4.35)	13.7 (8.5, NE)	[37]

ORR: objective response rate; DoR: median duration of response; PFS: median progression-free survival; OS: median overall survival; HR: hazard ratio; CI: confidence interval; NR: not reached; NE: not estimable; ^∗^follow-up at least 13 weeks; ^∗∗^follow-up at least 6 months.

**Table 3:** Treatment-related adverse events of US FDA-approved PD-1/PD-L1 inhibitors in patients with urothelial carcinoma.

Target	Inhibitor name	Treatment-related adverse events	Immune-related adverse events
PD-1	Nivolumab	Fatigue, decreased appetite, nausea, musculoskeletal pain, diarrhea, rash	Pneumonitis, hepatitis, colitis, endocrinopathies, nephritis, renal dysfunction, encephalitis, rash
	Pembrolizumab	Fatigue, decreased appetite, nausea, musculoskeletal pain, diarrhea, rash, pruritus, constipation	Pneumonitis, hepatitis, colitis, endocrinopathies, nephritis, renal dysfunction
PD-L1	Atezolizumab	Fatigue, decreased appetite, nausea, urinary tract infection, pyrexia, constipation	Pneumonitis, hepatitis, colitis, endocrinopathies (thyroid disease, adrenal insufficiency, hypophysitis, type 1 diabetes), meningitis/encephalitis, pancreatitis, dermatitis/rash
	Durvalumab	Fatigue, decreased appetite, nausea, urinary tract infection, diarrhea, musculoskeletal pain, constipation, peripheral edema	Pneumonitis, hepatitis, colitis, endocrinopathies (thyroid disease, adrenal insufficiency, hypophysitis, type 1 diabetes), nephritis
	Avelumab	Fatigue, decreased appetite, nausea, urinary tract infection, musculoskeletal pain	Pneumonitis, hepatitis, colitis, endocrinopathies, nephritis, renal dysfunction

**Table 4:** Selected new or ongoing clinical trials of PD-1/PD-L1 inhibitors for the treatment of urothelial carcinoma.

NCT identifier	Interventions	Recruitment	Phases	Locations
NCT03113266	Anti-PD-1 monoclonal antibody	Recruiting	Phase 2	China
NCT03287050	Pembrolizumab/radiation	Not yet recruiting	Early phase 1	United States
NCT03240016	Pembrolizumab/abraxane	Not yet recruiting	Phase 2	United States
NCT02807636	Atezolizumab/carboplatin/gemcitabine/cisplatin/placebo	Recruiting	Phase 3	Globe
NCT02853305	Pembrolizumab/cisplatin/carboplatin/gemcitabine	Recruiting	Phase 3	Globe
NCT03219775	Nivolumab/ipilimumab	Recruiting	Phase 2	Germany
NCT02500121	Pembrolizumab/placebo	Recruiting	Phase 2	United States
NCT02450331	Atezolizumab	Recruiting	Phase 3	Globe
NCT03115801	Atezolizumab/radiation	Recruiting	Phase 2	United States
NCT03244384	Pembrolizumab/clinical observation/biomarker analysis	Recruiting	Phase 3	United States
NCT02451423	Atezolizumab dose level 1/dose level 2/dose level 3	Recruiting	Phase 2	United States
NCT02897765	NEO-PV-01/nivolumab/adjuvant	Recruiting	Phase 1	United States
NCT02845323	Nivolumab + urelumab/nivolumab monotherapy	Recruiting	Phase 2	United States
NCT02736266	Pembrolizumab	Recruiting	Phase 2	Italy
NCT03237780	Atezolizumab/eribulin mesylate/biomarker analysis	Not yet recruiting	Phase 2	United States
